# Interactions between the neuromodulatory systems and the amygdala: exploratory survey using the Allen Mouse Brain Atlas

**DOI:** 10.1007/s00429-012-0473-7

**Published:** 2012-11-13

**Authors:** Andrew Zaldivar, Jeffrey L. Krichmar

**Affiliations:** 1 Department of Cognitive Sciences, University of California, Irvine, USA; 2 Department of Computer Science, University of California, Irvine, USA

**Keywords:** Neuromodulatory systems, Neuroinformatics, mRNA in situ hybridization, Allen Mouse Brain Atlas, Amygdala, Gene expression

## Abstract

Neuromodulatory systems originate in nuclei localized in the subcortical region of the brain and control fundamental behaviors by interacting with many areas of the central nervous system. An exploratory survey of the cholinergic, dopaminergic, noradrenergic, and serotonergic receptor expression energy in the amygdala, and in the neuromodulatory areas themselves was undertaken using the Allen Mouse Brain Atlas. The amygdala was chosen because of its importance in cognitive behavior and its bidirectional interaction with the neuromodulatory systems. The gene expression data of 38 neuromodulatory receptor subtypes were examined across 13 brain regions. The substantia innominata of the basal forebrain and regions of the amygdala had the highest amount of receptor expression energy for all four neuromodulatory systems examined. The ventral tegmental area also displayed high receptor expression of all four neuromodulators. In contrast, the locus coeruleus displayed low receptor expression energy overall. In general, cholinergic receptor expression was an order of magnitude greater than other neuromodulatory receptors. Since the nuclei of these neuromodulatory systems are thought to be the source of specific neurotransmitters, the projections from these nuclei to target regions may be inferred by receptor expression energy. The comprehensive analysis revealed many connectivity relations and receptor localization that had not been previously reported. The methodology presented here may be applied to other neural systems with similar characteristics, and to other animal models as these brain atlases become available.

## Introduction

Neuromodulatory systems, composed of relatively small nuclei of neurons, are located in the sub-cortical region of the brain and control fundamental behaviors through interactions with broad areas of the nervous system (Briand et al. 2007; Krichmar 2008). These systems have distinct neurotransmitters, which include norepinephrine, dopamine, serotonin, and acetylcholine, and distinct sources of those neurotransmitters. When a biological organism experiences an important event in the environment, the activation of the neuromodulatory system contributes to the organism’s ability to commit an action accordingly. These actions include mitigating responses to risks, rewards, attentional effort, and novelty. Thus, it is important to understand the underlying structure of these neuromodulatory systems as it plays a role in higher-order cognition and in an organism’s survival.

The nuclei of many neuromodulatory systems have neurons that are the origins of a specific neurotransmitter. Cholinergic neurons, which originate in the basal forebrain, project to the cortex, amygdala, and hippocampus. Basal forebrain cholinergic neurons appear to modulate attention and optimize information processing (Baxter and Chiba 1999). Cholinergic neurons also originate in the brainstem pedunculopontine and laterodorsal tegmental nuclei and have projections to the amygdala, basal forebrain, and the ventral tegmental area (Semba and Fibiger 1992; Holmstrand and Sesack 2011). Dopamine (DA) is produced by two groups of cell bodies in the mesencephalon: the substantia nigra (SN) and the ventral tegmental area (VTA). The VTA projects to the nucleus accumbens (NAc) and is the pathway implicated in mediating reward related behaviors (Hyman et al. 2006). The SN is the source of dopamine in the basal ganglia. Both the SN and VTA project to the hippocampus (Scatton et al. 1980). Norepinephrine (NE) in the central nervous system is produced by the locus coeruleus, which projects to virtually all brain regions with the exception of basal ganglia (Berridge and Waterhouse 2003). The nucleus of the solitary tract (NTS) is another source of norepinephrine. There is a feedback loop in which the amygdala affects stress hormones, and then the stress hormones acts on the NTS, which then acts on the locus coeruleus, resulting in the release of NE in the amygdala. Norepinephrine activation in the amygdala helps to consolidate and modulate memory in other brain regions (McGaugh 2004). Serotonergic projections, which originate in the raphe nuclei of the brainstem, extend to almost all forebrain areas (Barnes and Sharp 1999; Hornung 2003). The cortex, ventral striatum, hippocampus, and amygdala are amongst the areas that are innervated by raphe efferents (Harvey 2003; Meneses and Perez-Garcia 2007). Because the sources of these neuromodulatory transmitters are well established, we may be able to infer their connectivity to other brain regions by examining the specific neurotransmitter receptor expression.

In recent years, analyzing gene expression data has become an effective means of investigating the structural organization, distribution and connectivity of the nervous system. Expression of genes is a process elucidated by the production of ribonucleic acid (RNA) transcripts within cells. In situ hybridization localizes these transcripts at cellular resolution, and allows researchers to determine whether a given gene is expressed in specific cells (Jin and Lloyd 1997). Using this technique, many elements important to neuronal processing, such as receptors, transporters, growth factors, etc., can be localized by detecting specific messenger ribonucleic acid (mRNA) sequences. There are several publicly accessible large-scale databases that explore mRNA and protein localization in the mammalian central nervous system to give other members of the scientific community access to use their datasets (Bota et al. 2003; Visel et al. 2004; Christiansen et al. 2006). Gene Expression Nervous System Atlas (GENSAT) is one such database that provides a collection of gene expression maps of the mouse brain and spinal cord (http://www.gensat.org) (Heintz 2004). GENSAT uses in situ hybridization as a screening process to visualize selected genes through enhanced green fluorescent protein (EGFP) expression on bacterial artificial chromosome (BAC) transgenic mice to generate an atlas of gene expression in the mouse brain (Heintz 2004).

In addition, there exist databases that provide insight on brain circuitry through use of existing data originating from pathway tracing and imaging techniques, such as the Brain Architecture Management System (BAMS) and the Collation of Connectivity on the Macaque Brain (CoCoMac). BAMS is an online knowledge management system that stores and infers relationships between data about the structural organization of mammalian central nervous system circuitry (http://brancusi.usc.edu/bkms/) (Bota et al. 2005). CoCoMac provides large-scale wiring diagram of the primate cerebral cortex for use in brain system analysis and computational modeling (http://cocomac.org/) (Kötter 2004). However, it is not always possible with these databases to specify the neurotransmitter associated with a projection. Moreover, these databases are not necessarily complete and may not contain experiments on connectivity between certain brain regions.

In this survey, we used a resource from the Allen Institute for Brain Science called the Allen Mouse Brain Atlas (ABA), a project that features an interactive, comprehensive, genome-wide image database of expression data for over 20,000 genes (Lein et al. 2007; Sunkin and Hohmann 2007; Ng et al. 2007). A combination of RNA in situ hybridization data, detailed Reference Atlases, and informatics analysis tools are integrated to provide a searchable digital atlas of gene expression (Lein et al. 2007). For each gene that has a successful probe, quantified expression energy can be extracted and analyzed.

Researchers have utilized the ABA in a variety of projects, from validating gene expression patterns seen in other species through various methodologies to encouraging new scientific discoveries in gene association, brain organization, behavior, and disease (Jones et al. 2009). For instance, French and Pavlidis (2011) used the ABA, along with BAMS, to show that gene expression signatures have a statistical relationship to connectivity (French and Pavlidis 2011). Other researchers have applied statistical component analysis techniques to gene expression data from the ABA to understand the genetic neuroanatomical architecture of the hippocampus (Thompson et al. 2008). One ABA study reviewed the expression of uridine diphosphate (UDP)-glucuronosyltransferase (UGT) and how it is distributed across neural areas involved with olfaction (Heydel et al. 2010). Despite the ABA having a wide application within neuroscience, there still remains a vast array of uncharted genomic data analysis (Jones et al. 2009).

The present study investigates the receptor expression energy among some of the classic neuromodulatory systems, and their interaction with the amygdala. The amygdala was chosen due to its importance in learning and memory, and because it is known to be strongly innervated by neuromodulators (Gallagher and Chiba 1996; McGaugh 2004, 2006). Since the neuromodulatory systems have localized sources and specific neurotransmitters, we suggest that connectivity relationships can be inferred by examining the expression energy of receptors specific to those neuromodulatory systems. For example, the expression energy of adrenergic receptors in the ventral tegmental area may imply that either the nucleus of the solitary tract or locus coeruleus has a direct projection to this dopaminergic system. Based on this assumption, an exploratory survey of the noradrenergic, cholinergic, dopaminergic, and serotonergic receptor expression energy in the amygdala and within anatomical origins of neuromodulatory systems was undertaken using the ABA. The comprehensiveness of the mouse ABA allowed us to better analyze and understand the organization of brain circuitry involved with classic neuromodulators. Using this methodology, the present study makes predictions regarding neuromodulator connectivity and receptor localization.

## Methods

The ABA is a standardized atlas of gene expression data from 56-day-old male C57BL/6J mice strains visualized by in situ hybridization (ISH) using a non-radioactive, digoxigenin-labeled anti-sense riboprobes. ABA provides an Application Programming Interface (API) to access gene expression energy in different anatomical regions of the mouse brain atlas (http://community.brain-map.org/confluence/display/DataAPI/Home). The API features a number of method calls that allow users to obtain data including high-resolution images, expression data from an experiment’s image series and 3D coordinates for atlas-annotated structures in 200-µm resolution.

To investigate expression energy volumes in the brain regions of interest, we wrote a Java program to access the ABA via calls to its API methods (data retrieved 28 February 2012). In particular, two ABA API methods were utilized for the survey: *Gene* API and *Expression Energy Volumes* API. The *Gene* API method was first used to obtain a listing of image series identification (ID) numbers given a list of genes (Table 1). The *Expression Energy Volumes* API returned gene expression energy data per voxel of the mouse brain for a given ID. The volume space returned by this method was divided into individual 200 µm 3D cubic sagittally arranged voxels on an (*x*, *y*, *z*) coordinate plane. Expression energy value, as defined in the ABA, represents the density of expression within a 200 µm voxel from grid data taken per image series ID (sum of expressing pixels/sum of all pixels in division) divided by the pixel intensity of expression in that voxel (sum of expressing pixel intensity/sum of expressing pixels). To account for different sized brain regions, expression energy values for a brain region were normalized by dividing the number of voxels in a brain region that contained expression energy by the maximum number of voxels for that given brain area. We made no attempt to normalize based on neuron size, but rather looked at receptor gene expression per anatomical region.

**Table 1:** List of neuromodulatory genes accessed from the ABA

Gene symbol	Gene name	ImageSeriesID	Receptor subtype
Adra1a	Adrenergic receptor, alpha 1a	74277700	G_q_-protein coupled
Adra1d	Adrenergic receptor, alpha 1d	69236807	G_q_-protein coupled
Adra2a	Adrenergic receptor, alpha 2a	70723343	G_i_-protein coupled
Adra2c	Adrenergic receptor, alpha 2c	70723357	G_i_-protein coupled
Adrb1	Adrenergic receptor, beta 1	77340494	G_s_/G_i_-protein coupled
Adrb2	Adrenergic receptor, beta 2	68744522	G_s_/G_i_-protein coupled
Chrm1	Cholinergic receptor, muscarinic 1	73907497	G_q_/G_s_/G_i_-protein coupled
Chrm2	Cholinergic receptor, muscarinic 2	70560343	G_i_-protein coupled
Chrm3	Cholinergic receptor, muscarinic 3	2095	G_q_-protein coupled
Chrm4	Cholinergic receptor, muscarinic 4	261	G_i_-protein coupled
Chrm5	Cholinergic receptor, muscarinic 5	74821591	G_q_-protein coupled
Chrna1	Cholinergic receptor, nicotinic, alpha polypeptide 1	75551465	Ligand-gated Na^+^/K^+^ cation channel
Chrna2	Cholinergic receptor, nicotinic, alpha polypeptide 2	75551460	Ligand-gated Na^+^/K^+^ cation channel
Chrna3	Cholinergic receptor, nicotinic, alpha polypeptide 3	69734723	Ligand-gated Na^+^/K^+^ cation channel
Chrna4	Cholinergic receptor, nicotinic, alpha polypeptide 4	1173	Ligand-gated Na^+^/K^+^ cation channel
Chrna5	Cholinergic receptor, nicotinic, alpha polypeptide 5	74821601	Ligand-gated Na^+^/K^+^ cation channel
Chrna6	Cholinergic receptor, nicotinic, alpha polypeptide 6	75551461	Ligand-gated Na^+^/K^+^ cation channel
Chrna7	Cholinergic receptor, nicotinic, alpha polypeptide 7	69237107	Ligand-gated Na^+^/K^+^/Ca^2+^ cation channel
Chrna9	Cholinergic receptor, nicotinic, alpha polypeptide 9	74821602	Ligand-gated Na^+^/K^+^ cation channel
Chrnb1	Cholinergic receptor, nicotinic, beta polypeptide 1	75831174	Ligand-gated Na^+^/K^+^ cation channel
Chrnb2	Cholinergic receptor, nicotinic, beta polypeptide 2	2097	Ligand-gated Na^+^/K^+^ cation channel
Chrnb3	Cholinergic receptor, nicotinic, beta polypeptide 3	79760470	Ligand-gated Na^+^/K^+^ cation channel
Drd1a	Dopamine receptor D1A	352	G_s_-protein coupled
Drd2	Dopamine receptor 2	357	G_i_/G_o_-protein coupled
Drd3	Dopamine receptor 3	69859867	G_i_/G_o_/G_s_-protein coupled
Htr1a	5-Hydroxytryptamine receptor 1A	79394355	G_i_/G_o_-protein coupled
Htr1b	5-Hydroxytryptamine receptor 1B	583	G_i_/G_o_-protein coupled
Htr1d	5-Hydroxytryptamine receptor 1D	71393418	G_i_/G_o_-protein coupled
Htr1f	5-Hydroxytryptamine receptor 1F	69859867	G_i_/G_o_-protein coupled
Htr2b	5-Hydroxytryptamine receptor 2B	71664130	G_q_/G_11_-protein coupled
Htr2c	5-Hydroxytryptamine receptor 2C	71393424	G_q_/G_11_-protein coupled
Htr3a	5-Hydroxytryptamine receptor 3A	74724760	Ligand-gated Na^+^/K^+^ cation channel
Htr3b	5-Hydroxytryptamine receptor 3B	68745408	Ligand-gated Na^+^/K^+^ cation channel
Htr4	5-Hydroxytryptamine receptor 4	69257849	G_s_-protein coupled
Htr5a	5-Hydroxytryptamine receptor 5A	71393430	G_i_/G_o_-protein coupled
Htr5b	5-Hydroxytryptamine receptor 5B	69257975	G_i_/G_o_-protein coupled
Htr6	5-Hydroxytryptamine receptor 6	69257981	G_s_-protein coupled
Htr7	5-Hydroxytryptamine receptor 7	71393436	G_s_-protein coupled

ImageSeriesID is an identification number for the experiment used to analyze gene expression

The (*x*, *y*, *z*) coordinates associated with an expression energy were mapped to brain structures using the annotated atlas provided with the ABA API main site (AtlasAnnotation200.sva). The annotated atlas provided an identifier for a brain structure at a given coordinate. This identifier was then compared with a separate dataset file (brainstructures.csv) to obtain the name of the brain region associated with the identifier. For instance, suppose an expression energy value was found at coordinate (40, 26, 26) for the dopamine receptor, Drd1a. The AtlasAnnotation200.sva would reveal that those coordinates corresponded to the informatics ID number 139, which the brainstructures.csv file would then indicate that the informatics ID represented the VTA of the mouse brain.

### Brain regions

**Fig. 1:**
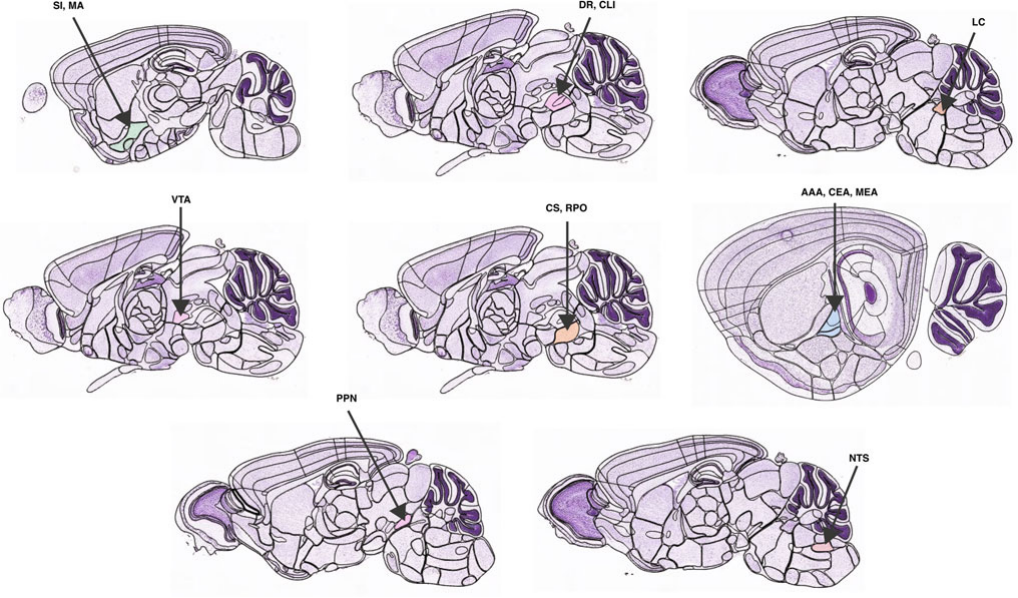
Image of reference atlas highlighting brain regions examined in the survey of neuromodulatory genes using the Allen Mouse Brain Atlas dataset. Brain regions studied include: dorsal raphe nucleus (DR), superior central nucleus raphe (CS), central linear nucleus raphe (CLI), nucleus raphe pontis (RPO), ventral tegmental area (VTA), locus coeruleus (LC), nucleus of the solitary tract (NTS), substantia innominata (SI), magnocellular nucleus (MA), pedunculopontine nucleus (PPN), anterior amygdalar area (AAA), central amygdalar nucleus (CEA) and medial amygdala nucleus (MEA). Image originally from the Allen Mouse Brain Reference Atlas (http://mouse.brain-map.org/static/atlas)

Expression data from the ABA were extracted from 13 different brain regions (Fig. 1). Ten of those regions are considered to be sources of neuromodulatory systems: noradrenergic (locus coeruleus, LC; nucleus of the solitary tract, NTS), cholinergic (substantia innominata, SI; magnocellular nucleus, MA; pedunculopontine nucleus, PPN), dopaminergic (ventral tegmental area, VTA), and serotonergic (dorsal raphe nucleus, DR; superior central nucleus raphe, CS; central linear nucleus raphe, CLI; nucleus raphe pontis, RPO) (Bhatia et al. 1997; Mesulam et al. 1983; Sodhi and Sanders-Bush 2004; Hornung 2003). The remaining three brain regions are in the amygdala (i.e., anterior amygdalar area, AAA; central amygdalar nucleus, CEA; medial amygdalar nucleus, MEA), which were chosen in this survey because of their strong bidirectional interaction with neuromodulatory systems (Bouret et al. 2003; Han et al. 1999; Lee et al. 2011; McGaugh 2004; Woolf and Butcher 1982). Note that the dopaminergic substantia nigra pars compacta was not included because it is thought to project primarily to the basal ganglia, an area not included in this study.

### Neuromodulatory genes

We performed a search in the ABA, using the *Gene* API, for all known neuromodulatory receptor genes, which included 5 dopaminergic, 16 serotonergic, 19 cholinergic, and 9 adrenergic receptors for a total of 49 different receptor types (Dani and Bertrand 2007; Hoyer et al. 2002; Ishii and Kurachi 2006; Lan et al. 2006; Nicholas et al. 1996). Of these 49, only 38 receptors were available for evaluation (Table 1). For example, some receptor genes, such as Drd4 and Drd5, were not available in the ABA, and thus, were not included in the present study. Although ABA data may extend from mouse brain tissue, all genes listed in Table 1 are orthologous to rat and human genes according to the Mouse Genome Informatics database (http://www.informatics.jax.org).

While the detection sensitivity for different probes may vary across mRNA species, the ABA has performed validation experiments to ensure consistent data quality and internal reproducibility (Lein et al. 2007; Lee et al. 2008). In every ISH run, a positive control slide was incubated with a Drd1a riboprobe and a negative control was incubated in hybridization buffer without that riboprobe (Lein et al. 2007). These slides were then used to determine whether data from the run would advance into their data analysis pipeline by qualitatively scoring the run as ‘Pass’ or ‘Fail’. In addition, an experiment was performed to replicate data across a series of days, using riboprobes generated in parallel through in vitro translation, which include Calb1, Calb2, Cst3, Dkk3, Gad1, Man1a, Plp1, Pvalb, and Nov (Lee et al. 2008). For each gene, an independently synthesized probe was hybridized on consecutive serial sections from the same brains over the span of 4 days, which maximizes comparability over time while minimizing other biological variability, including differential hapten incorporation in riboprobes, and batch reagent preparation variability. The results reported in Lee et al. (2008) demonstrate consistency of the ABA ISH platform.

In cases when multiple experiments (image series IDs) for a particular gene were found, we compared existing gene expression with the same search string and used the experiment that contained the highest expression energy data within brain regions of interest.

### GABA and glutamate genes

We also surveyed, exclusively within the SI and LC, the expression energy of GABA and glutamate receptors. We followed the same procedures as before when looking at neuromodulatory receptors. However, we instead searched and found all known GABA and glutamate receptors in the ABA via *Gene* API, which includes 17 GABA_A_, 2 GABA_B_, 4 AMPA, 5 kainate, 7 NMDA, and 7 mGluR receptors for a total of 42 different receptors. All GABA and glutamate genes are orthologous to rat and human genes according to the Mouse Genome Informatics database (http://www.informatics.jax.org).

## Results

Using the ABA, we conducted a comprehensive analysis of available neuromodulator receptor gene expression (see Table 1) in areas regarded as sources of neuromodulation, as well as the amygdala (see Fig. 1). The expression energy for all receptor subtypes, in which data was available in the ABA, was examined in all the brain regions of interest.

### Total expression and individual receptor subtypes

**Fig. 2:**
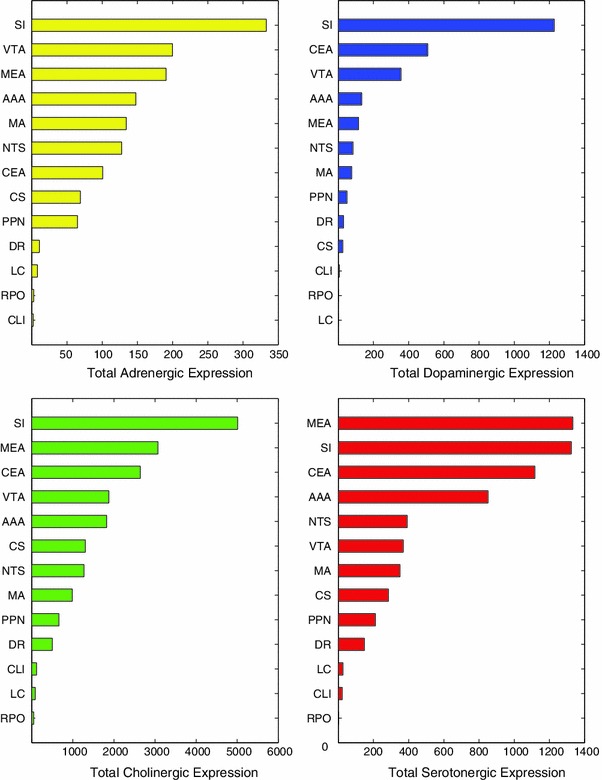
Total expression energy per brain region when combining all subtypes. Gene expression values for each subtype were collapsed into their respective neuromodulatory systems and separated by brain region. Brain regions were arranged from most (*top*) to least (*bottom*) amount of total expression

In the examined brain regions, expression energy of cholinergic receptors was much higher and expression energy of adrenergic receptors was much lower than that for dopaminergic and serotonergic receptors. Figure 2 shows the total expression energy for available adrenergic, cholinergic, dopaminergic, and serotonergic receptors from the ABA across the 13 brain regions examined (note the different scale on the *x*-axes of Fig. 2). Each bar in Fig. 2 represents gene expression energy when combining all receptor subtypes per region. Brain regions were ranked and arranged based on total expression in Fig. 2, with the brain region having the highest expression energy at the top bar of each plot.

The SI of the basal forebrain, amygdala (AAA, CEA, and MEA), and the VTA had relatively high levels of receptor expression energy. The SI had the highest receptor expression energy of all neuromodulatory regions tested, implying that this region of the basal forebrain is strongly innervated by all neuromodulatory systems (see Fig. 2). The amygdala closely followed SI in terms of overall neuromodulatory receptor expression energy, but expression energy in the amygdala differed based on neuromodulatory receptor type and amygdala subregions. For example, MEA had the highest adrenergic, cholinergic, and serotonergic receptor expression energy among the amygdala regions. However, the CEA had the most dopaminergic receptor expression energy. Similar to the SI, the VTA, which contains dopaminergic neurons, displayed high expression energy for all neuromodulatory receptors.

Somewhat surprisingly, the LC and raphe nuclei (DR, CS, CLI, and RPO), which are sources of norepinephrine and serotonin, respectively, did not have high expression energy of neuromodulatory receptors relative to the other regions examined (see Fig. 2). Because the expression energy was normalized by area, this difference should not be due to the smaller size of these brain regions.

**Fig. 3:**
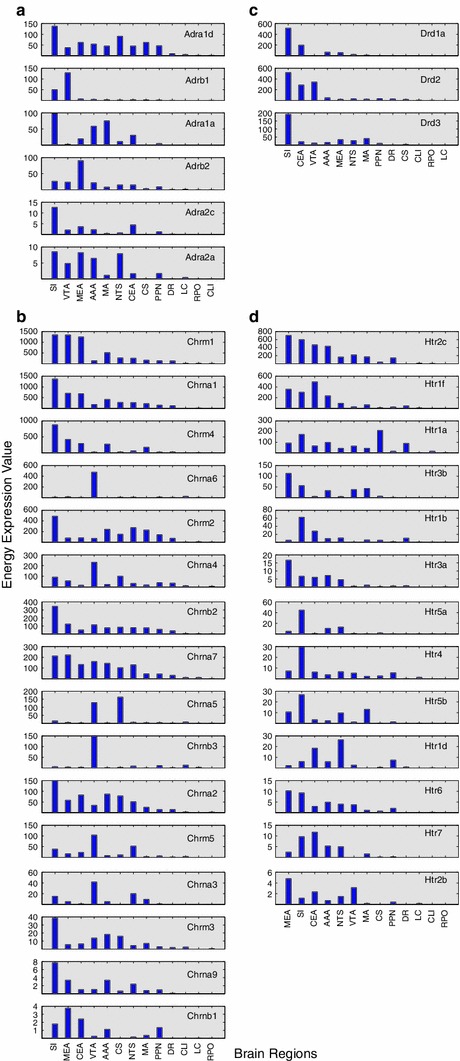
Expression of individual receptor subtypes across all neuromodulatory systems. Charts were grouped by neuromodulatory systems; **a** adrenergic, **b** cholinergic, **c** dopaminergic, and **d** serotonergic. Subtypes within each system were arranged from most (*left*) to least (*right*) amount of expression along the *x*-axis. Brain regions were ordered from most (*top*) to least (*bottom*) amount of total expression energy for each neuromodulator. The *y*-axis shows the expression energy for a given gene. Note that the *y*-axis scale varies for visualization purposes

Different brain areas had distinct patterns of receptor subtype expression. Expression energy for individual receptor subtypes across all neuromodulatory systems are shown in Fig. 3. Subtypes were sorted by expression per neuromodulatory system with the top charts having the highest expression. Within each neuromodulatory system, the arrangement of brain regions from left to right on each chart was based on their overall expression as in Fig. 2. It is apparent that the distribution of gene expression per subtype from one brain region to another was not uniform (Fig. 3). However, looking at individual expression energy helps identify receptor subtypes that contribute to the total expression of a particular brain region being described in Fig. 2.

The expression profile of SI, for example, which has the highest receptor expression energy among all for neuromodulatory regions (Fig. 2), may be influenced by select subtypes within neuromodulatory systems. For instance, within the adrenergic receptors, Adra1d and Adrb2 made up for a large portion of the expression energy found in SI, while the remaining four adrenergic receptors did not contribute nearly as much (Fig. 3a). The cholinergic system, which had the most receptor subtypes, was dominated by the expression of the muscarinic subtypes Chrm1, Chrm2 and Chrm4, and the nicotinic Chrna1 (Fig. 3b). Even the dopaminergic system, having the fewest receptor subtypes, had differing receptor expression, with Drd1a and Drd2 having much higher expression value in SI than Drd3 (Fig. 3c). Lastly, serotonergic receptors Htr2c, Htr1f, Htr1a, and Htr1b described most of the total expression energy in SI with comparatively lower contribution from the other subtypes (Fig. 3d).

Among the neuromodulatory sources, VTA also displayed higher overall receptor expression energy compared to other regions. In general, many of the subtypes that have noticeably high expression energy in the SI also have high energy in the VTA (Fig. 3). The main difference we observed was the muscarinic receptor (Chrm2), the nicotinic (Chrna4, Chrna6, Chrnb3), and the dopaminergic Drd2 receptor expression was higher in VTA than in SI (compare Fig. 3b with c).

**Fig. 4:**
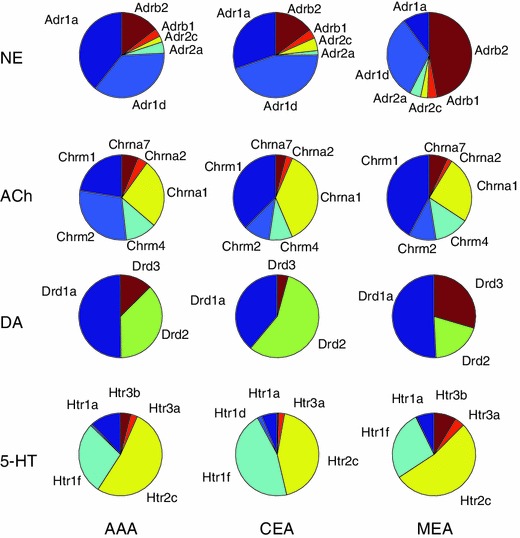
Distribution of gene expression within the different amygdala areas. Each *column* represents a different amygdala region (*AAA* Anterior amygdalar area, *CEA* central amygdalar area, *MEA* medial amygdalar area). Each *row* represents the distribution of expression for a particular neuromodulatory system. The amount of gene expression is relative to the slice size in each pie chart

Different regions of the amygdala have distinct patterns of neuromodulatory receptor expression energy. The neuromodulatory receptor expression energy found in the amygdala, which was among the highest of the brain regions studied in this survey, differed based on the neuromodulatory system (Fig. 2), amygdalar subregion, and by receptor subtypes (Fig. 3). For ease of visualization, pie charts were used to illustrate how receptor subtypes were distributed within the different amygdala areas (Fig. 4). Figure 4 revealed a similar distribution set of prominent gene expression across the amygdala areas with similar proportions. In the adrenergic system, Adra1a was highly expressed in the CEA and AAA, but lower in the MEA. In contrast, Adrb2 had higher expression energy in MEA than in AAA or CAE (Fig. 4, first row). The nicotinic receptor Chrna1 and the muscarinic receptor Chrm1 were more highly expressed across all the amygdala areas in comparison to other nicotinic and muscarinic receptors, though it is interesting to note that Chrm2 had relatively higher expression in the AAA as compared to CEA and MEA (Fig. 4, second row). Dopamine and serotonin receptors also showed differences in receptor expression energy across the amygdala. Drd2 and Htr1f contributed most strongly to the expression found in the CEA, whereas Drd1a and Htr2c contributed most strongly to the expression found in the AAA and MEA regions (Fig. 4, third and fourth row).

### Hierarchical clustering analysis

**Fig. 5:**
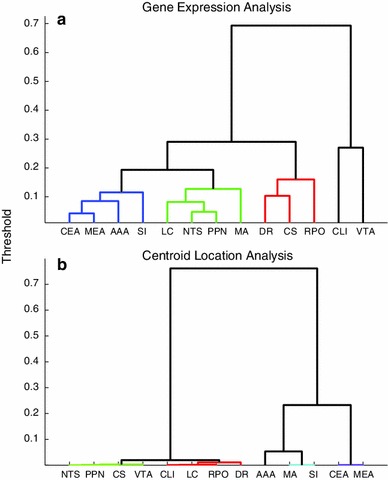
Hierarchical cluster of gene expression and location of brain region. **a** The dendrogram was derived from the expression of selected genes. **b** The dendrogram was derived from the *x*, *y*, *z* coordinates of brain area centroid given in the reference atlas. The dendrograms were generated using a Euclidean distance metric. The cutoff for generating the different clusters was set to 0.19 for (**a**) and 0.02 for (**b**), which broke the hierarchical cluster into four separate constitutes, denoted by their different coloring scheme

To illustrate the relationship between neuromodulatory receptor expression energy and brain region, hierarchical cluster analyses were performed for expression energy and anatomical location. A hierarchical clustering analysis is a commonly used exploratory technique to handle a large set of data whose interrelationships are elusive and not fully understood. The cluster analysis assigned subsets of gene expression data into groups based on the similarity in their expression patterns (Fig. 5a), and based on the location of the brain regions examined (Fig. 5b). A hierarchy of groupings can emerge using this methodology, and such analyses have previously shown relationships between biological function and anatomical location (Gerstein and Jansen 2000).

To perform the receptor expression energy cluster analysis, a vector of the total expression across the 38 genes was constructed for each of the 13 brain regions. The pairwise distance between these vectors were calculated using Euclidean distance. To create the dendrogram in Fig. 5a, an Unweighted Pair Group Method with Arithmetic Mean (UPGMA) was calculated based on the Euclidean distance metric. Threshold values in Fig. 5a represented the computed distance and linkage between brain regions. The cutoff for determining clusters was set to a threshold of 0.19 to yield three separate clusters, denoted by their different coloring scheme in Fig. 5a.

To examine the relationship between gene expression and anatomical location, a separate hierarchical cluster analysis was conducted using the centroid location for all of the 13 brain regions (Fig. 5b). The procedure was identical to the gene expression hierarchical cluster shown in Fig. 5a, except that a vector of the (*x*, *y*, *z*) coordinates from the reference atlas file (AtlasAnnotation200.sva) was used for clustering instead of gene expression data. The threshold for determining clusters was set to 0.02 to yield four clusters, as in Fig. 5b.

The clusters shown in Fig. 5 suggest several relationships between neuromodulatory receptor expression and anatomical location. The amygdala (AAA, MEA, CEA) and the SI formed a tight cluster (Fig. 5a, blue) in gene expression, as well as anatomically (Fig. 5b, cyan and purple). The SI and basal forebrain are located near the amygdala (see Fig. 1) and like the amygdala contain high overall neuromodulatory receptor expression energy (see Figs. 2, 3). LC and NTS, which contain noradrenergic neurons (McGaugh 2004; Samuels and Szabadi 2008), formed a tight cluster both in terms of gene expression and to a slightly lesser extent anatomically [Fig. 5a (green), b (green and red)]. There was also tight clustering among the raphe nuclei, the source of serotonin in the CNS [Fig. 5a (red), b (red and green)].

There were a few receptor expression energy clusters that did not match their anatomical cluster counterpart or did not form a strong cluster based on expression. For instance, the cholinergic sources SI and MA (Dani and Bertrand 2007; Ishii and Kurachi 2006; Nicholas et al. 1996) did not cluster together based on expression energy, though their distance apart from each other is still relatively small (Fig. 5a, blue and green). However, they are found in neighboring regions of the brain (see Fig. 1) and thus clustered together based on their centroid location (Fig. 5b, cyan). Perhaps the SI and MA not clustering together may be due in part to their proportionally higher expression energy across all four neuromodulatory systems in the SI as compared to MA (see Figs. 2, 3). The dopaminergic region (VTA) and the CLI of the raphe nucleus brain region did not fall within a cluster below the threshold when analyzing gene expression (Fig. 5a). However, in the anatomical cluster analysis, the VTA clustered together with all the raphe regions, PPN, and NTS (see Fig. 5, green and red), as they are located beside each other (Fig. 1).

### GABA and glutamate receptor distribution across SI and LC

One of our main findings was that the SI of the basal forebrain had high receptor expression energy for all four neuromodulatory systems (Fig. 2). In contrast, the LC had the lowest overall expression energy across the receptors examined (Fig. 2).

To see if high expression energy in SI and low expression energy in LC existed for receptors other than neuromodulators, we measured the expression energy of GABA and glutamate receptors in the SI and LC (see “GABA and glutamate genes”). We performed the same analysis as before for acquiring expression energy and generating the total expression of neuromodulatory systems found in the SI and LC (see “Total expression and individual receptor subtypes”) with these GABA and glutamate receptors.

**Fig. 6:**
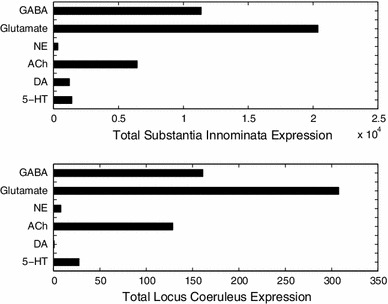
Total expression energy for GABA, glutamate, and neuromodulatory receptors across the substantia innominata (*top*) and locus coeruleus (*bottom*). Expression energy from neuromodulatory receptors is the same as in Fig. 2

We found that, similar to the profile of neuromodulatory receptors, the SI had very high expression energy of GABA and glutamate receptors, while the LC was low. Figure 6 shows the total expression energy for GABA and glutamate across the SI (top) and LC (bottom). For ease of visualization, in Fig. 6 we also included the total expression energy of adrenergic, cholinergic, dopaminergic, and serotonergic receptors from Fig. 2. The values in each bar in Fig. 6 represent the accumulated amount of expression energy when combining all subtypes per region. Note that there is a much higher order of magnitude in expression found in the SI compared to LC (Fig. 6).

Interestingly, we noticed a proportional relationship between the receptor expression found in the SI and LC. Though receptor expression in SI was much higher than in LC, the relative distribution of expression between GABA, glutamate, adrenergic, cholinergic, dopaminergic, and serotonergic receptors had very similar profiles to the LC, with glutamate receptors displaying the highest amount of expression, followed by GABA, acetylcholine, and serotonin (Fig. 6). This implies that the LC region has proportionally lower receptor expression energy when compared to SI, and other brain regions in this study. Since the receptor expression energy was normalized over region size (see “Methods”), this lower overall receptor expression energy level reflects a unique property of the LC region.

### Contrast between ABA data and prior in situ hybridization mRNA rat experiments

Although not exhaustive, neuroinformatics web resources such as the Gene Expression Nervous System Atlas (GENSAT) and the Neuroscience Information Framework (NIF) provide an accessible way to obtain gene expression data from various experiments (Heintz 2004; Gardner et al. 2008; Müller et al. 2008). We compared and contrasted data reported from the ABA to results from studies retrieved from these resources.

Table 2 shows the relative expression level in the brain regions of interest per receptor subtype. This was accomplished by first querying NIF using all genes listed in Table 1. NIF returned results from GENSAT that contained gene expression information from the mouse brain based on bacterial artificial chromosomes (BACs) experiments. However, because BAC experiments measure the relative rates of transcription for each gene, it is thereby not a direct measurement of mRNA accumulation. As such, in addition to the BAC expression data, GENSAT provides background literature, primarily from rat experiments, that measure localized mRNA using ISH, which GENSAT uses to correlate with their results. We utilized this feature to collect prior literature on gene receptor expression localization and intensities.

**Table 2:**
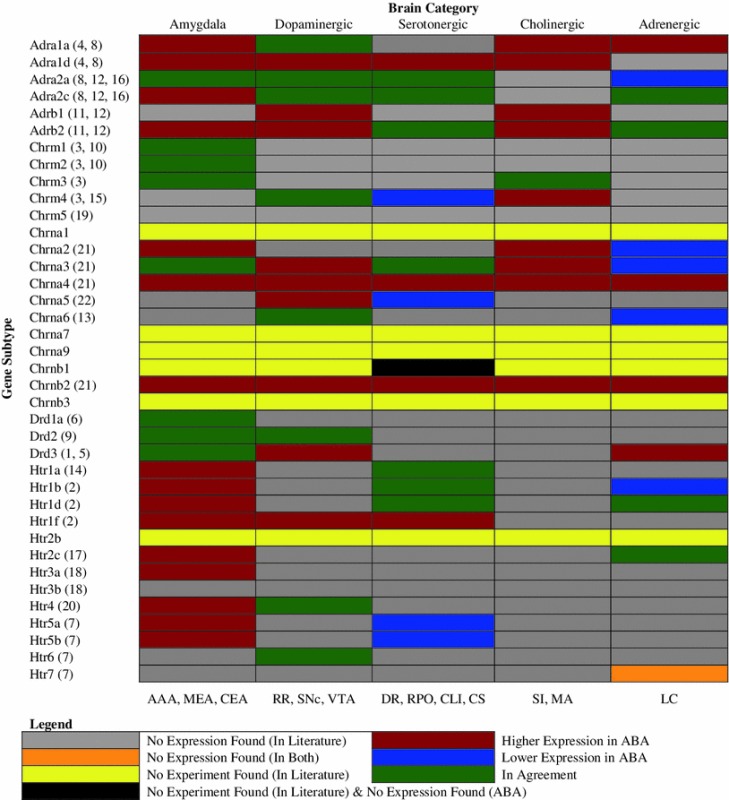
Comparison between expression levels in the brain regions of interest per subtype found in rat literature and ABA

Data from previous studies taken from: (1) Bouthenet et al. (1991), (2) Bruinvels et al. (1994), (3) Buckley et al. (1988), (4) Day et al. (1997), (5) Diaz et al. (1995), (6) Fremeau et al. (1991), (7) Kinsey et al. (2001), (8) McCune et al. (1993), (9) Mengod et al. (1989), (10) Narang (1995), (11) Nicholas et al. (1993), (12) Nicholas et al. (1996), (13) Novere et al. (1996), (14) Pompeiano et al. (1992), (15) Pompeiano et al. (1994), (16) Scheinin et al. (1994), (17) Sugaya et al. (1997), (18) Tecott et al. (1993), (19) Vilaró et al. (1990), (20) Vilaró et al. (2005), (21) Wada et al. (1989), (22) Wada et al. (1990)

Altogether, twenty-six papers were retrieved from GENSAT to compare and contrast gene receptor expression with the ABA in Table 2. With the exception of two receptors (Htr3a and Htr3b) coming from mouse literature, and six not having any prior literature found in GENSAT (Chrna1, Chrna7, Chrna9, Chrnb1, Chrnb3, Htr2b), all remaining receptors from Table 1 were available in GENSAT and feature rat brain in their experiment. As such, it should be noted that Table 2 is an indirect comparison of species to species receptor expression. However, all experiments retrieved from GENSAT document localization of mRNA using ISH.

Once literature was acquired, classification of expression level in the prior studies was taken directly from the wording in the corresponding reference. For example, some studies stated relative values (high, moderate, low), while others created tables using symbols (−, +, ++, +++) to denote the density of expression from in situ hybridization analysis. Classification of expression level in the present ABA study was based on the relative expression energy within a brain category. Expression energy less than the 33rd percentile was classified as low expression, moderate expression was between the 33rd and 66th percentiles, and above the 66th percentile was considered highly expressed. The 13 brain regions were condensed into 5 categories: Amygdala (AAA, MEA, CEA), Dopaminergic (VTA), Serotonergic (DR, RPO, CLI, CS), Cholinergic (SI, MA, PPN), and Adrenergic (LC, NTS) neuron regions. To determine the energy of expression, the average expression across these categorized brain regions was computed, and then percentiles were calculated across each gene in each category. If the expression of a gene (row) in a brain category (column) from the ABA coincided with previous work, then we considered the comparison to be in agreement, and the green entries in Table 2 denoted this. If the expression in the ABA was classified higher than in prior experiments, the table entry was colored red. Blue denoted lower expression in the ABA than in prior studies. Gray entries in the table represent expression data not found in previous studies, while yellow entries represent experiments not conducted in the literature. In the case where there was no expression found, but experiments conducted, in both the literature and ABA, entries were flagged in orange. Black entries represent a unique case where, for a given gene, no data was found in the ABA, but no experiment conducted in the literature.

In general, the comprehensiveness of the ABA revealed information that was previously unreported (Table 2, gray and yellow entries), and reported higher receptor expression in the amygdala and basal forebrain across all neuromodulatory systems than in previously reported studies (Table 2, red entries).

### Network visualization and connectivity

In order to better analyze complex systems of interaction, Pajek, a software package designed for examining large networks (Batagelj and Mrvar 1998), was used to visualize potential connectivity relationships between brain regions based on expression data from the ABA. We make the assumption that given a neuromodulatory source, such as VTA, we can infer the strength of a projection to a target area from that source based on the receptor expression energy (e.g., by looking at the overall dopaminergic expression energy in a target region).

**Fig. 7:**
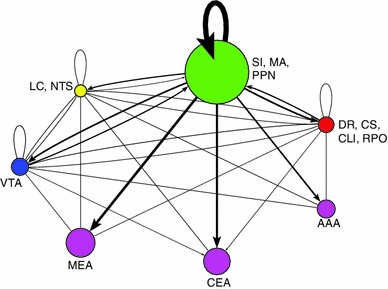
Network model showing overall expression of neuromodulatory receptors and their implied neuromodulatory projections to target areas. *Vertices* represent brain regions that are either standalone (AAA, CEA, MEA) or are combined regions (sources of neuromodulators). *Directed arcs* represent projections going to and coming from a source. The *pointed-arrow* indicates the target location and the *non-arrow end of the arc* indicates the origin. The thickness of each arc, as well as the size of vertices, is proportional to the amount of expression found in the target location. *Colors* were used for visualization purposes, similar to Figs. 2 and 3

Figure 7 shows the overall relationship among the neuromodulatory systems along with its interactions with the amygdala. Nodes corresponded to either a class of neurotransmitter source (e.g., ACh from SI, MA, and PPN) or the different regions of the amygdala, which were recipients of neuromodulation. Directional arcs represented inferred projections from a neuromodulatory system to a target brain area. The thickness of each arc was proportional to the amount of receptor expression energy found in the target region. The diameter of each node represented the total amount of receptor expression energy in that brain region. For example, the cholinergic receptor expression energy in MEA was much higher than serotonergic, as can be seen in Fig. 7 by the thickness of the arc (compare the arc extending from green node to MEA with the arc extending from the red node to MEA). All networks from Pajek were rendered using the circular layout; all other parameters were set to default. For ease of visualization, the amounts of receptor expression energy were scaled down, dividing the amount of receptor energy expression by 100.

Expression energy emanating from the cholinergic system is overwhelmingly the highest, followed by serotonergic, dopaminergic, and adrenergic (Fig. 7). All neuromodulatory systems project heavily to the cholinergic system as compared to other brain regions (Fig. 7 green node). The rest of the projections remained relatively low, though there may be an indication that serotonin projects more heavily to AAA compared to other amygdala areas (Fig. 7).

In addition to looking at the overall neuromodulatory connectivity network, we examined the influence of receptor subtypes on the different brain regions. Families of receptors were categorized in the following way: α (Adra1a, Adra1b, Adra2a, Adra2c) versus β (Adrb1, Adrb2) adrenergic receptors; muscarinic (Chrm1, Chrm2, Chrm3, Chrm4, Chrm5) versus nicotinic (Chrna1, Chrna2, Chrna3, Chrna4, Chrna5, Chrna6, Chrna7, Chrna9, Chrnb1, Chrnb2, Chrnb3) cholinergic receptors; D1 (Drd1a) versus D2 (Drd2, Drd3) dopaminergic receptors; and serotonin receptors that produce an inhibitory response (Htr1a, Htr1b, Htr1d, Htr1f, Htr5a, Htr5b) versus serotonin receptors that produce an excitatory response (Htr2b, Htr2c, Htr3a, Htr3b, Htr4, Htr6, Htr7).

**Fig. 8:**
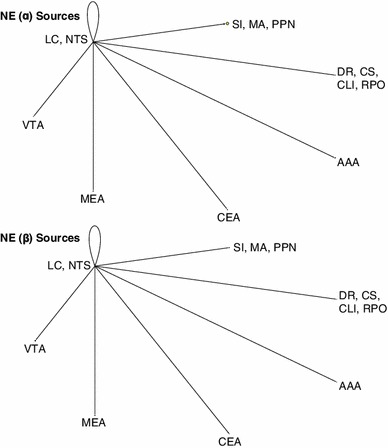
Network model comparison between the expression energy of α and β adrenergic receptors

**Fig. 9:**
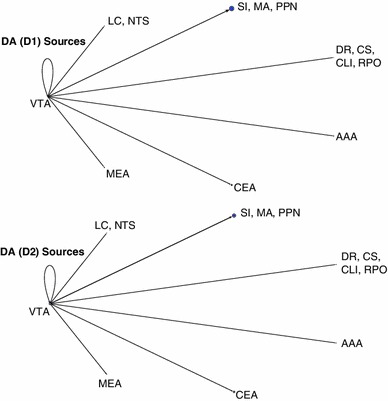
Network model comparison between the expression energy of muscarinic and nicotinic cholinergic receptors

**Fig. 10:**
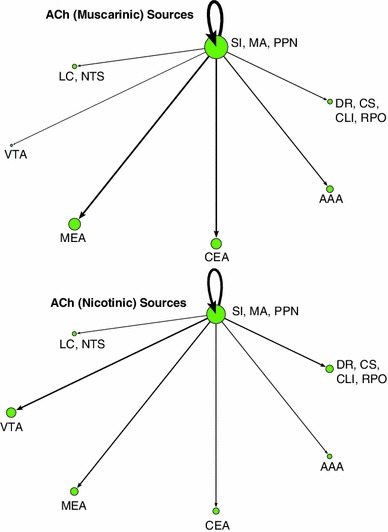
Network model comparison between the expression energy of D1 and D2 family dopamine receptors

**Fig. 11:**
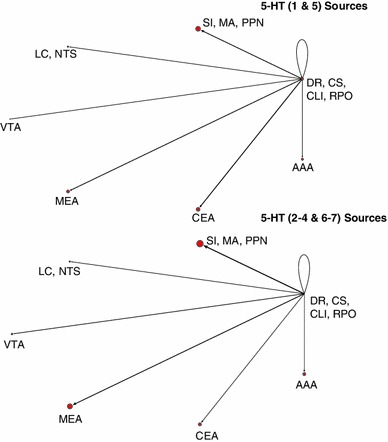
Network model comparison between the expression energy serotonin receptors that produce an inhibitory response (Htr1 and HTR5) and serotonin receptors that produce an excitatory response (Htr2, Htr3, Htr4, Htr6 and Htr7)

In general, different families of receptors had noticeable differences in how they are distributed across different brain regions (see Figs. 8, 9, 10, 11). For comparison purposes, the layout, arc thickness, and node diameter proportions were scaled down, dividing the amount of receptor energy expression by 1,000 for Figs. 8, 9, 10, 11. Expression energy from α-adrenergic receptors (Fig. 8, top) was more prevalent in cholinergic regions, as well as in the anterior amygdalar area (AAA), and within itself compared to β-adrenergic receptors (Fig. 8, bottom), which had a stronger influence on dopaminergic areas. Both the D1 and D2 dopamine families had a strong influence on the regions associated with acetylcholine and the CEA (Fig. 9); however, the D2 family of receptors expressed more within dopaminergic sources compared to D1 (Fig. 9, bottom). Muscarinic acetylcholine expression (Fig. 10, top) was higher than nicotinic expression in the amygdala (MEA, CEA), while nicotinic receptors (Fig. 10, bottom) were more strongly expressed in the dopaminergic areas. As for the serotonergic receptors, the amount of expression was roughly the same for the inhibitory and excitatory HTR families (Fig. 11).

## Discussion

Using the ABA, we conducted an exploratory survey of receptor expression energy in the classical neuromodulatory systems (i.e., cholinergic, dopaminergic, noradrenergic, serotonergic) within anatomical origins of these neuromodulatory systems and in the amygdala. These systems are somewhat unique in that the sources of the neurotransmitters are localized to small subcortical nuclei. The present study examined neuromodulatory receptor expression energy in the amygdala, which is thought to be a major target of neuromodulation, and within the sources of neuromodulation themselves (McGaugh 2004, 2006; Gallagher and Chiba 1996). Based on these assumptions, we were able to infer the targets of these neuromodulatory systems using receptor gene expression data from the ABA.

Although the present study was an exploratory survey of specific neuromodulatory receptor expression, several findings emerged from the study that could have functional implications: (1) Cholinergic receptors are overwhelmingly higher expression in the neuromodulatory nuclei than in the other classic neuromodulatory systems. Figures 2 and 7 show that the expression of cholinergic receptors is an order of magnitude higher than serotonin and norepinephrine, and much higher than dopamine. (2) The level of adrenergic expression was surprisingly small in all the brain areas tested. Moreover, the amount of neuromodulatory expression within the locus coeruleus was very low compared to other regions. Interestingly, the NTS, which is another source of noradrenergic neurons, displayed comparatively moderate expression energy of all neuromodulatory receptors. (3) The SI and VTA appear to be hubs, or ‘rich clubs’ of neuromodulation (van den Heuvel and Sporns 2011). In particular, the SI had the highest expression of all four neuromodulatory receptors compared to the other brain regions examined. (4) The amygdala is another hub of neuromodulation, with high receptor expression energy from all 4 neuromodulatory classes. Interestingly, SI is an anatomical neighbor of the amygdala making this anatomical region a neuromodulatory hub. (5) Lastly, the comprehensive ABA allowed the present survey to fill in many gaps in our knowledge of receptor expression using ISH. To the best of our knowledge, many of the results in the present study have not been reported previously in the rodent brain, as can be seen by the gray cells of Table 2.

It should be noted that our comparisons and interpretations may be influenced by a number of factors beyond the scope of this survey, including (1) differences in detection sensitivity between different mRNA species, which cannot be ruled out despite the ABA performing validation experiments, ensuring consistent data quality and internal reproducibility (see “Neuromodulatory Genes”), (2) not all receptor subtypes could be analyzed for some systems. For example, D4 and D5 were not present in the ABA, (3) mRNA may be transcribed, but not translated, into functional receptor proteins, and (4) the expression energy for a particular receptor may not necessarily be located at the synapse, or could be located pre-synaptically or post-synaptically (Feuerstein 2008; Gilsbach and Hein 2008; Wonnacott 1997). These cautionary remarks do not necessarily invalidate the present results, but they serve as a reminder that these factors should be considered and possibly investigated in future experiments using other methods, such as Western blots, to verify the present findings (Tebbenkamp and Borchelt 2010).

The completeness of the ABA allowed us to observe interesting patterns of neurotransmitter receptor expression energy, which may supplement current anatomical knowledge on neuromodulatory systems. Many of these expression patterns had not been previously reported (Table 2, gray and yellow entries). The amygdala (AAA, CEA, MEA), SI, and VTA showed the highest receptor expression energy of the regions examined (Fig. 2). The pattern of expression, for the most part, was similar within neuromodulator classes and among anatomical regions (compare Fig. 5a to b). Within an anatomical region, such as the amygdala, distinct patterns of receptor expression were observed across subregions (Fig. 4).

Bearing in mind that literature retrieved from GENSAT to compare and contrast receptor expression energies with the ABA in Table 2 originates primarily from rat studies (with the exception of Htr3a and Htr3b); our ABA survey suggests that the amygdala tended to show higher expression of neuromodulatory receptors than previously reported (McGaugh 2004; Han et al. 1999; Meneses and Perez-Garcia 2007; Haber et al. 1995) (Table 2, Amygdala column).

Among the prominent gene expression in the amygdala (Fig. 4), Chrm1, Chrm2, and the dopaminergic receptors were in agreement with literature findings (Narang 1995; Buckley et al. 1988) (Table 2). The rest, which includes Adra1d, Adrb2, Htr1f, Htr2c, and Htr3a has higher expression energy in the ABA than what was previously reported (Nicholas et al. 1996; Day et al. 1997; Goldman et al. 1986; Bruinvels et al. 1994); (Pompeiano et al. 1994). Though there were a few genes that did not have abundant expression yet were in agreement with literature data (Adra2a, and Chrna3), the remaining genes were either considered to have more expression than has been found, or no data was available for comparison (Table 2, Amygdala column).

Our findings for neuromodulatory receptor expression energy in the midbrain area, where dopaminergic neurons are found, in many places agreed and disagreed with previous work (Table 2, Dopaminergic column). In particular, we found that all of the α-adrenoreceptors, along with Chrna6, Chrnb3, Drd2, Htr4, and Htr6 were all in agreement with studies that have also shown expression from these receptors in the midbrain region (Day et al. 1997; Novere et al. 1996; Deneris et al. 1989; Vilaró et al. 2005; Kinsey et al. 2001).

The raphe nuclei, which are a source of serotonergic neurons, had fairly low expression energy overall (Fig. 2), and this expression was in agreement with several other studies (Table 2, Serotonergic column). More specifically, Adra2a, Adra2c, Adrb2, Chrna3, Htr1a, Htr1b, and Htr1d, had low-to-moderate expression energy in the present ABA and other studies (McCune et al. 1993; Scheinin et al. 1994; Nicholas et al. 1996). However, several receptor genes showed higher expression in the ABA than was previously reported (Adra1d, Chrna4, Chrnb2, Htr1f), as well as some receptor genes that displayed lower expression in the ABA than stated in prior literature (Chrm4, Chrna5, Htr5a, Htr5b). Still, we were not able to find data on many genes, with one gene in particular (Chrnb1) not found in both the literature and ABA data set (Table 2, Serotonergic column, gray, yellow and black entries).

Conversely, our findings in the basal forebrain (especially the SI), a source of cholinergic neurons, which showed the highest amount of expression out of all the brain regions in this study (Fig. 2), had very little agreement with literature data (Table 2, Cholinergic column). It has been reported that there are efferent projections of the adrenergic and serotonergic systems into the basal forebrain (Holmstrand and Sesack 2011; Samuels and Szabadi 2008; Hornung 2003). However, the present study suggests a significantly larger neuromodulatory innervation of the basal forebrain, compared to other neuromodulatory regions, than previously reported. Adrenergic (Adra1a, Adra1d, Adrb1, Adrb2) and cholinergic (Chrm4, Chrna2, Chrna3, Chrna4, Chrnb2) receptors were classified as having higher expression in the ABA than in previous studies. However, no information in literature data was found for the remaining receptors (Table 2, Cholinergic column, gray entries). That, along with the substantially high receptor expression energy found in the SI in this survey, suggests that future studies should focus on this region.

The locus coeruleus and the NTS, which are major sources of noradrenergic neurons, had several genes that were classified as having lower expression energy in the ABA than other studies (Fig. 2; Table 2, Adrenergic column). Adra2a, Chrna2, Chrna3, Chrna6, and Htr1b were all reported to have moderate-to-high expression in the locus coeruleus, yet the data in the ABA suggest lower expression (McCune et al. 1993; Scheinin et al. 1994; Nicholas et al. 1996) (Table 2, Adrenergic column). Furthermore, Htr7 was the only gene that had no data in both the ABA and literature (Table 2, Adrenergic column, orange entry). In terms of agreement, only the Adra2c, Adrb2, Htr1d, and Htr2c receptors, which had low-to-moderate energy of expression, match former findings (McCune et al. 1993; Scheinin et al. 1994; Nicholas et al. 1996; Goldman et al. 1986; Wada et al. 1989; del Toro et al. 1994; Bruinvels et al. 1994; Mengod et al. 2006; Pompeiano et al. 1994). All other genes were not found in literature (Table 2, Adrenergic column, gray entries).

The completeness of the Allen Brain Atlas for the mouse brain is a rich source for exploratory studies and made the present neuroinformatics study possible (Lein et al. 2007; Jones et al. 2009). Our study, which took advantage of the somewhat unique structure of the neuromodulatory systems, was able to create a connectivity map from the sources of neuromodulation to their receptor targets in the amygdala and the neuromodulatory nuclei (see Figs. 7, 8, 9, 10, 11). The study revealed connectivity relations and receptor localization that had not been reported previously. The pattern of expression varied across regions, not just in the level of expression, but also by receptor subtypes. These variations may have functional and anatomical implications.

Our survey of the ABA showed interesting and novel relationships between the neuromodulatory systems and the amygdala. The comprehensive mouse atlas provided by the ABA allowed us to form a more complete picture of these interactions than seen previously. The methodology presented here may be applied to other neural systems with similar characteristics, and to other animal models as their brain atlases become available.
